# 

**Published:** 1913-06-07

**Authors:** 


The American Association for the Advancement of Research by Women has awarded the Ellen Richards Prize of £200 to Dr. Ida Smedley (Mrs. MacLean) for her work on the bio-chemical synthesis of fatty acids. When the prize was last awarded in 1909 the successful candidate was also an Englishwoman, Dr. Florence Buchanan.

### Appointments.

Mr. J. F. Dobson, M.S., F.R.C.S., has been elected honorary surgeon to the Leeds General Infirmary in the place of Mr. Littlewood, retired, and Mr. S. W. Daw, F.R.C.S., assistant honorary surgeon.

Owing to the increased work placed upon the Local Government Board for Scotland by the National Insurance Act, Dr. Ernest Watt has been appointed an additional medical inspector of the Board. Miss Mary J. Menzies has been appointed lady medical inspector in place of Miss Elizabeth McVail, who has resigned that position.

At a meeting of the Special Committee for the election of honorary medical officers held under the chairmanship of Mr. Alfred Simpson, Dr. Ernest Bosdin Leech, M.D., B.C. (Cantab.), D.P.H. (Vict.), M.R.C.P. (London), M.R.C.S. (Eng.), was elected to the office of honorary assistant physician at the Manchester Royal Infirmary.

